# An Account of Two Cases in Which a Large Quantity of Laudanum Was Swallowed

**Published:** 1815-05

**Authors:** R. Jones

**Affiliations:** Surgeon. *Chancery-lane*


*For the Medical and Physical Journal.*

### *An Account of Two Cases in which a large Quantity of Laudanum was Swallowed;* by Mr. R. Jones.

OBSERVING, in your last Journal, a case of Recovery from the Effects of Opium, I am induced to send you a short account of two cases wherein my assistance was lately called, with the results attending.

On the morning of the 8th of December last, I was called to —— Wedgwood, No. 1, Chichester Rents; who had taken about half an ounce, or between that and an ounce, of Tinct. Opii. I saw him in about an hour after: his friends about him, having in the mean time discovered what he had done, sent to a neighbouring chemist, who administered to him an emetic, which had no effect. I took with me an ounce of Vin. Antimon. which I diluted with water, and at intervals got him to swallow. The patient was drowsy, and particularly chilly, arising, of course, from decreased arterial action. I directed the messenger who came for me, to give the patient half a pint of vinegar, as soon as he got back; and hence commenced my perplexity, for the opium, acid, or both conjointly, had acted on the stomach so much as a sedative, that, though he had taken first a strong emetic from the chemist, an ounce of antimonial wine, two scruples of ipecacuanha, and more than a quart of warm water, yet there was no disposition to vomit. However, after rumbling and tossing him about for near an hour, we were fortunate enough to see the desired effect. Similar vexation occurred to Mr. Rowe. As the evacuation of the stomach is the first consideration, ought we to delay that object by using acids, which, though they may coagulate or render inert the opium solution for a while, yet manifestly paralyse our first efforts? I am inclined to think we ought not, as acids themselves are, under such circumstances, strongly sedative. We well know that an inebriated man, when his stomach is inclined or actually rejects its contents, will instantly find it staid or relieved by the use of acids. This man recovered, but was weak and languid for some days after; and the discipline he underwent seemed to have cured him of all suicidical notions—he appeared repentant.

On Thursday the 16th of February last, I was called to Susanna Chettle, at No. 1, Carey-street, who had taken an ounce of Tinct. Opii. The viol, which was found by her, (and a tea-cup, out of which she had drank it,) could not have contained more. She was discovered about noon quite insensible; they immediately sent for me. I found her dying, dying, as I supposed from apoplexy, having upon her the apoplectic snorting; but upon examination, and inquiring a little into particulars, added to the discovery of the viol, I had no doubt of the cause, but which was now too late to be remedied. She died in a few minutes after I arrived. She was about 60 years of age, given to drink, and had been inebriated the day before, which, no doubt, facilitated the action of the poison.

There is one thing which I wish to call the attention of your readers to in this and similar cases arising from vegetable poisons; which, in the absence of circumstantial evidence or direct proof, shall distinguish them from apoplexy. This is the state of the pupil. I have found it more or less contracted from the effect of vegetable poisons, and always according to the degree in which they acted. I have found it the same in people insensibly drunk: whereas, in effusions upon the brain, or into its ventricles, or concussion, I have invariably found it dilated.* I am aware that the belladonna, when applied to the eye, will dilate the pupil; but will it have the same effect when internally taken? However, the symptoms arising from the effects of these poisons, it is well known, vary in almost every individual; but it would be of great importance if this can be established as a distinguishing symptom. R. JONES, Surgeon.

*Chancery-lane; March 9th.*

---

*For the Medical and Physical Journal.*

### *Essay on the Functions of the Nervous System;* by Mr. C. W. Smerdon.

*(Continued from p. 280.)*

IN no instance, I will contend, is the analogy between galvanism and nervous energy so striking, as in the process of secretion: on the one side, we may instance the de-

---

* I was called last summer to a young woman, about 18 or 19 years of age, who, without previous indisposition, fell down deprived of all sense, and died a few hours after. Upon opening her head the next day, I found the vessels of the dura and pia mater a little turgid with blood; but the cause of her death was found to be owing to an effusion of blood into the right lateral ventricle, which was plugged with a firm coagulum, and had expelled the water through its communication into the left, which also was filled with mixed blood and water. The pupil here was exceedingly dilated, proving that the same effect is produced by pressure from within the brain, as we usually find it from that without.

composition

